# Outcomes of patients with ST-segment myocardial infarction admitted during the COVID-19 pandemic: A prospective, observational study from a tertiary care center in Germany

**DOI:** 10.1007/s00059-021-05058-7

**Published:** 2021-08-17

**Authors:** M. Rattka, C. Winsauer, L. Stuhler, K. Thiessen, M. Baumhardt, T. Stephan, W. Rottbauer, A. Imhof

**Affiliations:** Department of Cardiology, Ulm University Medical Centre, Albert Einstein Allee 23, 89081 Ulm, Germany

**Keywords:** SARS-CoV‑2, STEMI, Epidemiology, Emergency medical services, Mortality, SARS-CoV‑2, ST-Strecken-Hebungs-Infarkt, Epidemiologie, Medizinische Notfalldienste, Mortalität

## Abstract

**Background:**

Since the beginning of the SARS-CoV‑2 outbreak, healthcare professionals reported that patients admitted with ST-segment myocardial infarction (STEMI) were in worse condition compared to STEMI patients admitted before the outbreak. However, data on their outcomes are sparse.

**Methods:**

We conducted a prospective, observational, cohort study of STEMI patients admitted during the COVID-19 pandemic from March 21, 2020 to July 31, 2020 (COVID-19 group). Clinical outcomes, 30-day mortality, and reasons potentially related to a delay in patient presentation were assessed and compared with STEMI patients admitted between November 1, 2019 and March 20, 2020 (pre-COVID-19 group).

**Results:**

A total of 124 patients were enrolled, comprising 57 patients in the pre-COVID-19 group and 67 patients in the COVID-19 group. Significantly more patients in the COVID-19 group had a time to first medical contact of greater than 24 h. Additionally, those admitted during the pandemic had a significantly lower left ventricular ejection fraction (LVEF), worse thrombolysis in myocardial infarction (TIMI) flow, received circulatory support significantly more often, and had a significantly higher 30-day mortality. Furthermore, significantly more patients stated that “information by the media” made them hesitate to contact the emergency medical services as soon as possible.

**Conclusion:**

Here, we show that STEMI patients admitted during the COVID-19 pandemic had significantly prolonged times to first medical contact, were in worse condition at admission, and had an increased 30-day mortality. Additionally, we found that “information by the media” made patients during COVID-19 hesitate to contact the emergency medical services. Consequently, public health strategies have to be developed to avoid potential excess mortality of STEMI patients during the pandemic.

**Supplementary Information:**

The online version of this article (10.1007/s00059-021-05058-7) contains supplementary material, which is available to authorized users.

## Abstract (de)

**Hintergrund:**

Seit Beginn des Ausbruchs der SARS-CoV-2-Pandemie berichteten medizinisch Tätige, dass mit einem ST-Strecken-Hebungs-Infarkt (STEMI) aufgenommene Patienten in einem schlechteren Zustand waren als STEMI-Patienten mit stationärer Aufnahme vor dem Ausbruch. Allerdings ist die Datenlage zu deren Ergebnissen spärlich.

**Methoden:**

Es wurde eine prospektive Beobachtungs-Kohortenstudie an STEMI-Patienten mit stationärer Aufnahme während der COVID-19-Pandemie vom 21. März bis 31. Juli 2020 (COVID-19-Gruppe) durchgeführt. Klinische Ergebnisse, 30-Tage-Mortalität und potenziell mit einer verspäteten Patientenvorstellung assoziierte Gründe wurden untersucht und mit STEMI-Patienten verglichen, die zwischen 1. November 2019 und 20. März 2020 stationär aufgenommen worden waren (Prä-COVID-19-Gruppe).

**Ergebnisse:**

In die Studie wurden 124 Patienten eingeschlossen, 57 in die Prä-COVID-19-Gruppe und 67 in die COVID-19-Gruppe. In der COVID-19-Gruppe betrug die Zeit bis zum medizinischen Erstkontakt bei signifikant mehr Patienten länger als 24 h. Darüber hinaus wiesen während der Pandemie aufgenommene Patienten eine deutlich niedrigere linksventrikuläre Ejektionsfraktion (LVEF) und einen schlechteren TIMI-Wert („thrombolysis in myocardial infarction“) auf, sie erhielten öfter Kreislaufunterstützung und hatten eine deutlich höhere 30-Tage-Mortalität. Außerdem gaben deutlich mehr Patienten an, dass sie aufgrund der „Information durch die Medien“ gezögert hatten, schnellstmöglich den medizinischen Notfalldienst zu kontaktieren.

**Schlussfolgerung:**

Die Autoren zeigen, dass STEMI-Patienten mit stationärer Aufnahme während der COVID-19-Pandemie signifikant längere Zeiten bis zum medizinischen Erstkontakt aufwiesen, sich in schlechterem Zustand präsentierten und eine erhöhte 30-Tage-Mortalität aufwiesen. Außerdem stellten die Autoren fest, dass die „Information durch die Medien“ die Patienten zögern ließ, den medizinischen Notfalldienst zu kontaktieren. Folglich sollten Strategien des öffentlichen Gesundheitswesens entwickelt werden, um während der Pandemie eine potenzielle Übersterblichkeit von STEMI-Patienten zu verhindern.

Since the beginning of the coronavirus-19 (COVID-19) pandemic in December 2019, efforts have been undertaken to contain the spread of severe acute respiratory distress syndrome coronavirus 2 (SARS-CoV-2) and to minimize infection-related morbidity and mortality. It has been observed that not only the SARS-CoV‑2 infection itself can affect the medical care of the general population, but more indirect effects of the pandemic (such as lockdown, stay-at-home orders, and iatrophobia) can potentially lead to additional excess morbidity and mortality beyond the COVID-19 disease [1–3]. For patients suffering from acute cardiac events, physicians from around the world have reported on declining admission numbers during the pandemic [4–10]. Remarkably, this effect was not limited to epicenters of the pandemic, as it could also be demonstrated for regions less impacted by the virus [4, 5].

Among patients with acute heart disease, those with ST-segment myocardial infarction (STEMI) appear to be an especially vulnerable population. Their outcome is particularly influenced by the total ischemic time, which has been observed to be prolonged during the pandemic [6, 11]. In this context, cardiologists observed that STEMI patients admitted during the COVID-19 outbreak have higher serum troponin T levels, worse left ventricular systolic function, suffer more in-hospital complications, and have higher hospital mortality rates compared to rates before the pandemic [4, 12, 13]. However, at present, there are no prospective data on the outcome of STEMI patients who presented during the COVID-19 pandemic.

## Methods

### Study design and study population

In this prospective, single-center, observational cohort study, we included all patients with STEMI consecutively admitted between the day public restriction measures came into effect on March 21, 2020 and July 31, 2020, who were defined as the COVID-19 group. For the control group (pre-COVID-19 group), all STEMI patients admitted between November 1, 2019 and March 20, 2020 were assessed for eligibility retrospectively. Included patients had to be ≥ 18 years old, suffer from STEMI, and give written informed consent. The diagnosis of STEMI was made according to contemporary guidelines and STEMI patients underwent a percutaneous coronary intervention (PCI), as indicated by current recommendations [14]. All STEMI patients were monitored at our coronary care unit (CCU), as appropriate [14]. Those in critical condition were admitted to our intensive care unit (ICU), instead. The study complies with the Declaration of Helsinki and was approved by the local ethics committee (number of application and positive vote 250/20). This study adheres to the STROBE statement [15].

### Baseline data collection

Demographic, clinical, laboratory, and in-hospital outcome data were assessed. Blood samples were drawn at the time of hospital admission and during in-hospital stay for measurements of high-sensitivity cardiac troponin T (hsTnT) and NT-proBNP (ElectroChemiLumineszenz ImmunoAssay “ECLIA” Roche, Cobas 8000, Basel, Switzerland, Module e801 and e601) as part of the clinical routine. Following local standards, every patient admitted since March 21, 2020 has been tested for SARS-CoV‑2 by throat swab test (Sigma-Virocult with 2 ml Virocult medium, Check Diagnostics GmbH, Westerau, Germany) and analyzed by reverse transcription polymerase chain reaction (RT-PCR) at the local Institute for Virology. Left ventricular systolic function before dismissal was analyzed by automated echocardiographic quantification (EPIQ 7, Koninklijke Philips N.V., Eindhoven, The Netherlands, 2004). Heart failure symptoms were assessed according to the NYHA classification and symptoms of cardiac ischemia were defined by the CCS classification.

### Clinical follow-up and outcomes

After dismissal, depending on their clinical condition, patients were scheduled for outpatient clinic visits after 1 month, 3 months, and then every 6 months, as part of our clinical routine. If an outpatient clinic visit could not be performed, a home visit was offered to the patient. Echocardiography and blood sample measurements of hsTnT and NT-proBNP were conducted at the outpatient clinic visits at the discretion of the attending physician. Left ventricular systolic function was assessed by automated echocardiographic quantification (in-hospital: EPIQ 7, Koninklijke Philips N.V., Eindhoven, The Netherlands; outpatient visit: Butterfly IQ, Butterfly Network. Inc., Guilford, CT, USA). All patients were treated according to scientific guidelines.

### Outcomes

Outcomes assessed were heart failure symptoms as measured by NYHA class, degree of angina pectoris as measured by CCS class, left ventricular systolic function, serum NT-proBNP levels, and 30-day mortality. The follow-up period started with the day of admission due to STEMI. Additionally, we evaluated patient time from symptom onset to first medical contact (FMC) and assessed factors potentially related to a delay in admission using a five-item questionnaire (Supplementary Material 1; [3, 4, 16]). The questionnaire was completed during the initial hospitalization, during a telephone survey, or at the follow-up visit. Patients were free to answer or not answer the questions, as deemed appropriate.

### Statistical analysis

Continuous variables with normal distribution, assessed with the Kolmogorov–Smirnov test, were compared using the *t *test. Numeric variables not normally distributed were analyzed with the Mann–Whitney rank sum test and described as median and interquartile range (IQR). Categorical variables are described as absolute and relative values and analyzed using the chi-square test or Fisher’s exact test, as appropriate. The Kaplan–Meier estimator was used to assess the time to death, and groups were compared using the Cox proportional hazards model. A two-sided *p *value of less than 0.05 was considered statistically significant. Due to the explorative nature of this study, all results from statistical tests have to be interpreted as hypothesis generating. An adjustment for multiple testing was not carried out. Statistical assessment was performed by SPSS Statistics 25 software (Version 2017, IBM, Armonk, NY, USA).

## Results

### Patient characteristics

**Fig. 1:**
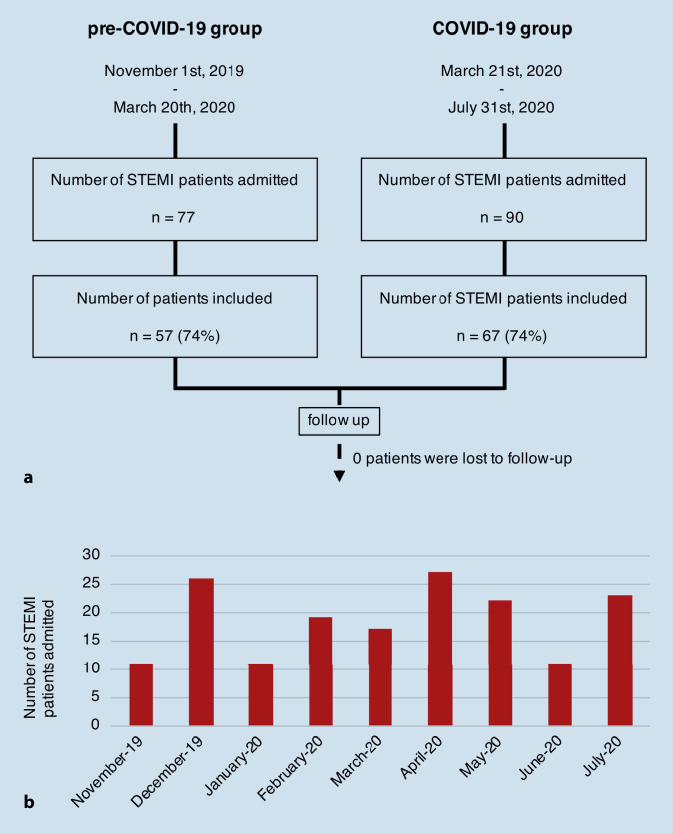
Patient allocation and number of STEMI patient admissions. Between November 1, 2019 and July 31, 2020, 167 patients with STEMI were admitted to our tertiary care center. Of those, 124 patients met the inclusion criteria and gave written informed consent, and were enrolled in our study (**a**). The number of STEMI admissions did not decrease after the first COVID-19-positive patients had been identified in March 2020 (**b**)

Between November 1, 2019 and July 31, 2020, 167 patients with STEMI were treated at our tertiary care center, including 90 patients who were admitted during the COVID-19 pandemic and 77 patients admitted before the outbreak. In total, 57 patients (74%) admitted before the outbreak and 67 patients (74%) admitted after measures of social restriction, which were implemented in Germany on March 21, 2020, gave written informed consent and were included in our study (Fig. 1a). The STEMI patients included in the study had a mean age of 65 years with 77% being male. At least one cardiovascular risk factor was present in 89.5% of the patients. Admissions of patients with STEMI per month were similar before and during the COVID-19 period (Fig. 1b). No patients tested positive for SARS-CoV‑2. A comparison of baseline characteristics of the COVID-19- and pre-COVID-19 groups did not show any significant differences (Table 1).

**Table 1:** Demographic characteristics

	Pre-COVID-19 *n* = 57	COVID-19 *n* = 67	*p*
Age (years)	63 ± 13	65 ± 14	0.411
Male sex	47 (82)	48 (72)	0.156
Arterial hypertension	36 (63)	40 (60)	0.694
Diabetes mellitus	15 (26)	18 (27)	0.945
Obesity	7 (12)	14 (21)	0.202
Family history	18 (32)	13 (19)	0.119
CKD	12 (21)	18 (30)	0.451
History of TIA/stroke	5 (9)	3 (4)	0.468
OSAS	1 (2)	5 (7)	0.217
COPD	3 (5)	2 (3)	0.660
Smoker	28 (49)	31 (46)	0.751

The data are mean ± standard deviation or absolute frequencies (%)

*CKD* chronic kidney disease, *TIA* transient ischemic attack, *OSAS* obstructive sleep apnea syndrome, *COPD* chronic pulmonary obstructive disease

### Clinical characteristics

Clinical, laboratory, and echocardiographic parameters were assessed in order to evaluate the effect of the COVID-19 outbreak on the clinical status of STEMI patients at admission. Assessment of the times to FMC revealed that significantly more patients admitted during the outbreak had a time to FMC greater than 24 h compared to the control group (pre-COVID-19: 5 out of 55 patients; COVID-19 14 out of 59 patients; *p* = 0.036). Remarkably, serum troponin T levels at admission were elevated in STEMI patients admitted during the COVID-19 pandemic. However, this was a strong but nonsignificant trend (pre-COVID-19: 266 [64, 1126]; COVID-19: 583 [158, 2165]; *p* = 0.064). Systolic left ventricular ejection fraction at admission was significantly reduced in the COVID-19 group compared to the pre-COVID-19 group (pre-COVID-19: 52% [46, 62]; COVID-19: 45% [40, 56]; *p* = 0.019). While there was no difference for culprit lesion, thrombolysis in myocardial infarction (TIMI) flow before (*p* = 0.039) and after PCI (*p* = 0.020) was significantly worse in STEMI patients admitted during the pandemic. Moreover, patients in the COVID-19 group had a significantly greater need for circulatory support (30%) during and after PCI compared to the pre-COVID-19 group (12%; pre-COVID-19: 7 out of 57 patients; COVID-19: 20 out of 66 patients; *p* = 0.016). Moreover, significantly more patients in the COVID-19 group had to be monitored at the ICU after PCI had been performed (pre-COVID-19: 40 out of 57 patients (70%); COVID-19: 63 out of 67 patients (94%); *p* < 0.001). The total duration of hospitalization was similar in both groups. Detailed results are displayed in Table 2.

**Table 2:** Clinical characteristics at baseline

	Pre-COVID-19 *n* = 57	COVID-19 *n* = 67	*p*
NYHA class (*n* = 114)			
I II III IV	29 (53) 19 (35) 4 (7) 3 (5)	4 (7) 2 (3) 6 (10) 47 (80)	< 0.001
CCS class (*n* = 114)			
0 I II III IV	4 (8) 1 (2) 3 (6) 5 (9) 40 (75)	6 (10) 2 (3) 3 (5) 2 (3) 48 (79)	0.700
Time to FMC (*n* = 114)			
≤ 24 h > 24 h	50 (91) 5 (9)	45 (76) 14 (24)	0.036
Systolic blood pressure (mm Hg; *n* = 109)	124 ± 26	119 ± 28	0.364
Diastolic blood pressure (mm Hg; *n* = 109)	71 ± 15	69 ± 19	0.627
Troponin T (ng/l; *n* = 115)	266 [64, 1126]	583 [158, 2165]	0.064
NT-pro BNP (pg/ml; *n* = 118)	354 [74, 1520]	532 [138, 3382]	0.258
LVEF (%) (*n* = 81)	52 [46, 62]	45 [40, 56]	0.019
Culprit lesion (*n* = 124)			
LAD LCX RCA	30 (53) 6 (10) 21 (37)	28 (42) 12 (18) 27 (40)	0.363
TIMI flow before PCI (*n* = 124)			
0 I II III	12 (21) 22 (39) 18 (31) 5 (9)	26 (39) 27 (40) 13 (19) 1 (2)	0.039
TIMI flow after PCI (*n* = 124)			
0 I II III	0 (0) 2 (4) 17 (30) 38 (66)	0 (0) 12 (18) 23 (34) 32 (48)	0.020
Circulatory support (*n* = 123)	7 (12)	20 (30)	0.016
Catecholamines (*n* = 123)	4 (7)	13 (20)	0.042
Mechanical (*n* = 123)	5 (9)	15 (23)	0.036
Monitoring at ICU (*n* = 124)	40 (70)	63 (94)	< 0.001
Duration of hospitalization (h; *n* = 120)	107 [88, 136]	110 [63, 138]	0.945

The data are mean ± standard deviation, median [IQR] or absolute frequencies (%)

*NYHA* New York Heart Association, *CCS* Canadian Cardiovascular Society, *FMC* first medical contact, *LVEF* left ventricular ejection fraction, *LAD* left anterior descending artery, *LCX* left circumflex artery, *RCA* right coronary artery, *TIMI* thrombolysis in myocardial infarction,* PCI* percutaneous coronary intervention, *ICU* intensive care unit

### Outcomes

After a median follow-up of 148 days [49; 235], there was no significant difference in left ventricular systolic function, NT-proBNP levels, mean NYHA class, and mean CCS class. However, symptoms of heart failure and cardiac ischemia improved in both groups compared to the index event. Importantly, 30-day mortality was significantly increased in STEMI patients admitted during the COVID-19 pandemic (pre-COVID-19: 6 out of 57 patients; COVID-19: 17 out of 67 patients; *p* = 0.034; Table 3).

**Table 3:** Outcomes

	Pre-COVID-19 *n* = 57	COVID-19 *n* = 67	*p*
30-day mortality (*n* = 124)	6 (11)	17 (25)	0.034
NYHA class (*n* = 99)			
I II III IV	25 (51) 18 (37) 3 (5) 3 (6)	19 (38) 20 (40) 6 (12) 5 (10)	0.491
CCS class (*n* = 99)			
0 I II III IV	36 (73) 8 (16) 2 (4) 1 (2) 2 (4)	38 (76) 3 (6) 2 (4) 2 (4) 5 (10)	0.415
LVEF (%; *n* = 84)	54 [46, 62]	50 [45, 59]	0.212
NT-pro BNP (pg/ml; *n* = 61)	392 [126, 793]	672 [235, 1495]	0.070

The data are median [IQR] or absolute frequencies (%)

*NYHA* New York Heart Association, *CCS* Canadian Cardiovascular Society, *LVEF* left ventricular ejection fraction

### Factors of reluctance to seek timely medical attention

Several outlets in scientific and public media have speculated that during the COVID-19 pandemic, patients refrain from seeking urgent medical attention despite suffering from severe symptoms, and, thereby, possibly worsen their own prognosis [3, 4]. Therefore, we evaluated potential factors delaying time to FMC during times of COVID-19. Interestingly, significantly more patients stated that “information by the media” made them hesitate to contact the emergency medical services as soon as possible (pre-COVID-19: 0%, 0 out of 48 patients; COVID-19: 11%, 5 out of 46 patients; *p* = 0.019). There was no significant difference for other factors such as fear, framing, and altruistic behavior (Table 4).

**Table 4:** Assessment of factors possibly delaying immediate admission

	Pre-COVID-19	COVID-19	*p*
Framing—pulmonary disease (*n* = 89)	5 out of 46 (11)	8 out of 43 (19)	0.302
Framing—musculoskeletal disease (*n* = 93)	11 out of 48 (22)	15 out of 45 (33)	0.263
Fear of contagion in-hospital (*n* = 96)	4 out of 48 (8)	9 out of 48 (19)	0.136
Altruistic behavior (*n* = 95)	2 out of 48 (4)	5 out of 47 (11)	0.227
Information by the media (*n* = 94)	0 out of 48 (0)	5 out of 46 (11)	0.019

The data are absolute frequencies (%)

## Discussion

To our knowledge, this is the first prospective, observational study on STEMI patients admitted during the COVID-19 pandemic. We observed that STEMI patients in the COVID-19 group had significantly longer times to FMC, a lower left ventricular ejection fraction at the initial presentation, a worse TIMI flow, and a significantly higher need of circulatory support. Additionally, they were admitted to the ICU significantly more often. This was associated with a significantly higher 30-day mortality. Remarkably, among STEMI patients in the COVID-19 group, “information by the media” seemed to be a decisive factor that potentially kept them away from hospital during the pandemic.

Since the beginning of the COVID-19 pandemic, healthcare professionals and cardiologists reported on the phenomenon of missing STEMI patients, which has been observed in both epicenters and non-epicenters of the viral outbreak [5, 6, 10]. It has been suggested that the external effects of the pandemic might keep patients with acute coronary syndrome from receiving urgent medical attention, and, thereby, worsen their prognosis [4, 10]. We, among others, noticed that STEMI patients admitted during the initial period of the pandemic appeared to be in poorer condition than before the outbreak. There have been several reports observing higher serum troponin T levels, worse left ventricular systolic function at admission, higher in-hospital complication rates, and higher in-hospital fatality rates [4, 11–13]. This was, in part, associated with a prolonged time from symptom onset to FMC [6, 11, 17, 18]. In our current study, STEMI patients in the COVID-19 group had a significantly lower left ventricular systolic function, worse TIMI flow, a greater need for circulatory support, and, consequently, had to be admitted to the ICU more often for prolonged monitoring. This substantiates previous reports. However, despite being in worse clinical condition, there was no difference in duration of hospitalization. This is most likely related to (1) the higher in-hospital mortality in the COVID-19 group, and (2) the physicians’ decision to discharge patients admitted during the pandemic early, which mitigates the risk of getting infected with SARS-CoV‑2 in hospital [16].

Next, to evaluate whether clinical patient characteristics of the index hospitalization are associated with a delay in admission, we assessed the time to FMC. We found that significantly more patients in the COVID-19 group had a time to FMC greater than 24 h. This indicates that even in regions that have been less impacted by the pandemic, STEMI patients suffered from prolonged delay times potentially affecting their prognosis negatively.

Several reasons have been presented to contribute to the phenomenon of missing and delayed STEMI admissions. Among others, fear of getting infected with SARS-CoV‑2 in hospital, misled altruistic behavior, framing issues, implementation of social distancing measures, and the influence of the media have been proposed to affect patient behavior [3, 4, 16]. For a better understanding of the causes keeping STEMI patients away from hospital, we asked patients included in our study about reasons that might have prolonged their time to FMC using a five-item questionnaire. In the COVID-19 group, 19% stated that they thought their symptoms would be lung-related rather than heart-related and 33% suspected muscular causes to be responsible for their suffering. Furthermore, 11% said that they did not want to overburden the healthcare system and 19% feared getting an infection while in hospital. However, these single factors by themselves did not differ significantly between the COVID-19 group and the pre-COVID-19 group. Intriguingly, significantly more people in the COVID-19 group (11%) than in the pre-COVID-19 group (0%) stated that “information by the media” made them hesitate to get in contact with the emergency medical services. Consequently, external factors (e.g., the media) rather than internal factors (e.g., fear, framing, and misled altruistic behavior) might be responsible for the decrease in STEMI admissions. Similarly, Wu et al. reported that, for England, STEMI patients admitted during the imposed lockdown period decreased in number and had longer times from call to hospital admissions compared to the post-lockdown phase [7]. This illustrates that external factors appear to have an adverse impact on the health behavior of STEMI patients during the COVID-19 pandemic and need to be minimized in the future.

Whether the COVID-19 pandemic has had an impact on the mortality of STEMI patients admitted during the outbreak has not been evaluated sufficiently to date. Reports from COVID-19 hotspots, such as Italy and China, noted a significant increase in in-hospital mortality of STEMI patients admitted since the beginning of the crisis [6, 13]. On the contrary, in less affected regions, no effect on in-hospital mortality was detected [7, 18–20]. A recent meta-analysis assessing the in-hospital mortality of STEMI patients admitted during the pandemic could not detect a significant difference [21]. However, prospective data on this topic are sparse. To shed more light on this issue, we prospectively investigated the 30-day mortality of STEMI patients admitted during the COVID-19 outbreak in Germany for the first time. Intriguingly, we found that mortality was significantly increased in STEMI patients admitted during the pandemic. Consequently, it seems that even in regions that have been struck less by the virus in the initial phase of the pandemic (such as Germany), STEMI patients admitted during the outbreak suffer a more unfavorable outcome compared to patients admitted before the pandemic. Our data suggest that this finding might be attributed to the significant delay in first medical contact and worse TIMI flow observed in STEMI patients admitted during the pandemic, which are both known to be independently associated with infarct size and excess mortality of STEMI patients [22–24].

In conclusion, it appears reasonable to suggest that indirect effects of the pandemic (e.g., information by the media or lockdown) kept patients from seeking urgent medical attention and, thereby, explains the observed excess mortality. However, our data are generated from a single hospital and, consequently, our results have to be interpreted as hypothesis-generating. Prospective, multicenter studies including a larger number of patients are necessary to verify our results.

### Limitations

As this is a prospective, observational study of the outcomes of STEMI patients admitted before and during the COVID-19 pandemic to a single center, it inherently has limitations. Although our university clinic is a high-volume center, since the beginning of the pandemic only a definite number of STEMI patients have been admitted, which limited the number of patients eligible for inclusion. Consequently, our results have to be interpreted as hypothesis-generating. Larger, prospective multicenter studies would be desirable to further assess this issue. Additionally, the follow-up period was significantly longer in the pre-COVID-19 group, which is attributable to the study design. Moreover, since the COVID-19 pandemic unexpectedly spread across the world, only patients in the COVID-19 group could be enrolled at baseline, and patients in the pre-COVID-19 group were recruited retrospectively. However, during data acquisition, the status of every patient included was able to be assessed.

## Conclusion

Our results demonstrate that STEMI patients admitted to a tertiary care center in a non-epicenter region during the initial phase of the COVID-19 pandemic had significantly prolonged times to first medical contact compared with patients admitted before the outbreak. This was associated with worse clinical condition at admission, worse TIMI flow, and increased 30-day mortality. Additionally, “information by the media” might have adversely influenced the patients’ health behavior. Consequently, public health measures must be implemented in the future management of the pandemic to avoid potential excess mortality. Multicenter studies are necessary to verify our results.

## Supplementary Information

Supplementary material 1: 5‑item questionnaire on factors possibly delaying immediate admission

## Declarations

All procedures performed in studies involving human participants or on human tissue were in accordance with the ethical standards of the institutional and/or national research committee and with the 1975 Helsinki declaration and its later amendments or comparable ethical standards. Informed consent was obtained from all individual participants included in the study.
