# Supplementary material for: Outcomes of patients with ST-segment myocardial infarction admitted during the COVID-19 pandemic: A prospective, observational study from a tertiary care center in Germany
Source: Herz. 2021 Aug 17;47(3):258–64. doi: 10.1007/s00059-021-05058-7 (PMC8369437; doi:10.1007/s00059-021-05058-7)
Supplement: Supplementary file 1 — Supplementary material 1: 5‑item questionnaire on factors possibly delaying immediate admission [file 59_2021_5058_MOESM1_ESM.pdf]

1. I thought my complaints could be related to a pulmonary disorder.
2. I thought my complaints could be related to a musculoskeletal disorder.
3. I feared getting infected in-hospital.
4. I thought my admission could overburden the medical personal or health system.
5. Information by the media made me reconsider my admission.

Every question could be answered by one of the following choices:

- Yes
- No
- Do not know / Do not want to answer

Supplementary material 1: 5-item questionnaire on factors possibly delaying immediate admission
